# Clinical treatment planning for kilovoltage radiotherapy using EGSnrc and Python

**DOI:** 10.1002/acm2.13832

**Published:** 2022-11-28

**Authors:** Mihails Nikandrovs, Brendan McClean, Laura Shields, Patrick McCavana, Luis León Vintró

**Affiliations:** ^1^ School of Physics University College Dublin Belfield Ireland; ^2^ Centre for Physics in Health and Medicine University College Dublin Belfield Dublin Ireland; ^3^ St. Lukes Radiation Oncology Network Dublin Ireland

**Keywords:** kilovoltage, Monte Carlo, orthovoltage, Python, treatment planning system

## Abstract

Kilovoltage radiotherapy dose calculations are generally performed with manual point dose calculations based on water dosimetry. Tissue heterogeneities, irregular surfaces, and introduction of lead cutouts for treatment are either not taken into account or crudely approximated in manual calculations. Full Monte Carlo (MC) simulations can account for these limitations but require a validated treatment unit model, accurately segmented patient tissues and a treatment planning interface (TPI) to facilitate the simulation setup and result analysis.

EGSnrc was used in this work to create a model of Xstrahl kilovoltage unit extending the range of energies, applicators, and validation parameters previously published. The novel functionality of the Python‐based framework developed in this work allowed beam modification using custom lead cutouts and shields, commonly present in kilovoltage treatments, as well as absolute dose normalization using the output of the unit. 3D user‐friendly planning interface of the developed framework facilitated non‐co‐planar beam setups for CT phantom MC simulations in DOSXYZnrc.

The MC models of 49 clinical beams showed good agreement with measured and reference data, to within 2% for percentage depth dose curves, 4% for beam profiles at various depths, 2% for backscatter factors, 0.5 mm of absorber material for half‐value layers, and 3% for output factors.

End‐to‐end testing of the framework using custom lead cutouts resulted in good agreement to within 3% of absolute dose distribution between simulations and EBT3 GafChromic film measurements. Gamma analysis demonstrated poor agreement at the field edges which was attributed to the limitations of simulating smooth cutout shapes. Dose simulated in a heterogeneous phantom agreed to within 7% with measured values converted using the ratio of mass energy absorption coefficients of appropriate tissues and air.

## 1 INTRODUCTION

Skin cancers, most commonly found in the head and neck region,^1^ are often treated with kilovoltage (kV) radiotherapy. Many patients undergo several courses of treatment in their lifetime with potential regions of dose overlap, and therefore it is important to know accurate 3D dose distributions of previous and current treatment plans to guide clinical decision making. Conventionally, kV radiotherapy dose calculations are performed with manual calculations of point doses in the water‐based geometry. This approach has several important limitations when compared to commercial treatment planning systems (TPS). First, it does not account for the presence of tissue inhomogeneity under skin surface in the region of clinical interest. It was previously shown that surface dose is reduced by up to 12.5% with the presence of underlying bone or 10.5% with shielding material such as lead.^2^, ^3^, ^4^, ^5^, ^6^, ^7^, ^8^ There are also significant absorbed dose differences between different tissue types due to the differences in mass energy absorption coefficients.^9^, ^10^, ^11^ Second, the use of an inverse square law correction, or similar, for an average source‐to‐surface distance in the treatment field is a crude mechanism in dealing with highly irregular surfaces present in the head and neck region. Third, custom‐lead cutouts and shields are often used in kV radiotherapy and their presence is only accounted for by using interpolated backscatter factors (BSF) from the published IPEMB data,^12^ assuming an equivalent square or circular field size. While this approach is a simple approximation for surface doses, it provides no dose distribution information at depth which would be helpful for clinical decision making. These limitations justify a need for a more accurate approach to kV treatment planning and dose calculations.

Monte Carlo (MC) calculations are accepted as a gold standard dose calculation algorithm in commercial TPS as they explicitly simulate radiation transport through any medium and account for all irregular geometries. To the best of our knowledge, there are no commercially available clinical TPS for kV radiotherapy. However, MC calculations have been extensively used in kV energy range research.^13^ Several studies have created MC models of kV radiotherapy units.^9^, ^14^, ^15^, ^16^, ^17^, ^18^, ^19^ Knoos et al.^14^ modeled 120 and 200 kV beams of the D3225 unit (Gulmay Medical Ltd, UK) and simulated two sample dose distributions on patient CT images. Chow et al.^19^ used MATLAB (The MathWorks Inc.) to create a TPS for small animal irradiations limited to a single energy of 225 kVp. Penchev et al.^16^ modeled WOmed T‐200 kV unit (WOLF‐Medizintechnik GmbH, Germany) and developed a graphical user interface to set up simulations for a range of dosimetric problems using phantoms and CT images. Their framework allowed modification of the applicator geometry but did not allow further beam modification, for example, using lead cutouts or shielding. The dose was reported as a relative dose and an absolute dose calculation approach was proposed.

This study focused on extending the work of the aforementioned authors to create and validate an MC model of the Xstrahl 200 kV unit (XSTRAHL Ltd, UK) along with 49 specific energy‐applicator combinations used in our facility. The beam model was extensively validated against reference data of percentage depth dose (PDD) curves, beam profiles, half‐value layers (HVL), BSFs, and radiation output factors, providing a better match than some previously published models. Python,^20^ being an accessible and commonly used coding language, was used in this work to develop a treatment planning interface (TPI), opening up the possibility of sharing the code with other users. This TPI introduced the ability to simulate custom lead cutouts and shields making it compatible with any kV treatment setup. Furthermore, an absolute dose calculation functionality was validated using gamma analysis and measurements in an end‐to‐end testing of the framework. User‐friendly 3D interactive treatment planning process allowed physicists without prior MC knowledge or experience to set up, run, and analyze MC simulation results making this framework easy to integrate into routine clinical practice.

## 2 METHODS AND MATERIALS

The work comprised of three parts. In the first part, an MC model of the kV unit was created and validated against reference data for a full range of clinically used applicators and beam energies. In the second part, Python applications were developed and tested, simplifying the process of creating simulation input files (CT‐based phantom and simulation parameters). In the third part, a tissue segmentation approach was developed and an end‐to‐end test of the workflow was conducted using custom‐lead cutouts on a water phantom as well as simulations of a heterogeneous phantom.

### 2.1 Kilovoltage unit model creation and validation

EGSnrc is a well validated and widely used general purpose code for MC simulations developed by National Research Council (NRC) of Canada, with radiotherapy specific applications: BEAMnrc and DOSXYZnrc.^21^ BEAMnrc was used in this work to create a model of the X‐ray tube inside the Xstrahl 200 kV unit as well as the applicator geometry for 25 applicators, which ranged from 20 to 50 cm source‐to‐surface distance (SSD), 2 cm circle to 20 × 20 cm^2^ field sizes and were both open and closed ended.

Monoenergetic electron beams with energies of 70, 100, 125, and 200 keV and 7 mm diameter were modeled with horizontal incidence onto the tungsten target (1 cm thick and 1 cm diameter) of the X‐ray tube at 30° to the photon beam axis. The resultant photon beam was filtered appropriately using one of the four filter combinations from Table 1. 1 × 10^9^ histories were used for all simulations. MC parameters and variance reduction techniques (VRTs) were implemented as recommended by BEAMnrc for low‐energy simulations.^22^ Directional Bremsstrahlung Splitting (DBS) was implemented with a splitting number of 2 000 resulting in uncertainties of less than 1% at all dose values of interest. All simulations were performed on a quad‐core hyperthreading PC Intel(R) Xeon(R) CPU W3520 @ 2.67GHz.

**TABLE 1:** Xstrahl 200 unit nominal beam energies, photon beam filtration present, and reference HVLs as beam quality specifiers

**kVp**	**Filtration (mm)**	**HVL**
70	2 Al	2.15 mm Al
100	0.1 Cu, 0.5 Al	4.25 mm Al
125	0.2 Cu, 2.5 Al	8.125 mm Al
200	0.4 Sn, 0.25 Cu, 1 Al	1.85 mm Cu

Abbreviation: HVL, half‐value layers.

Three BEAMnrc setup configurations were used in this work. In the first configuration, Phase Space (PHSP) files were generated at the exit plane of each applicator and were used to extract beam fluence in air and as inputs in DOSXYZnrc watertank and later CT phantom simulations.

**FIGURE 1:**
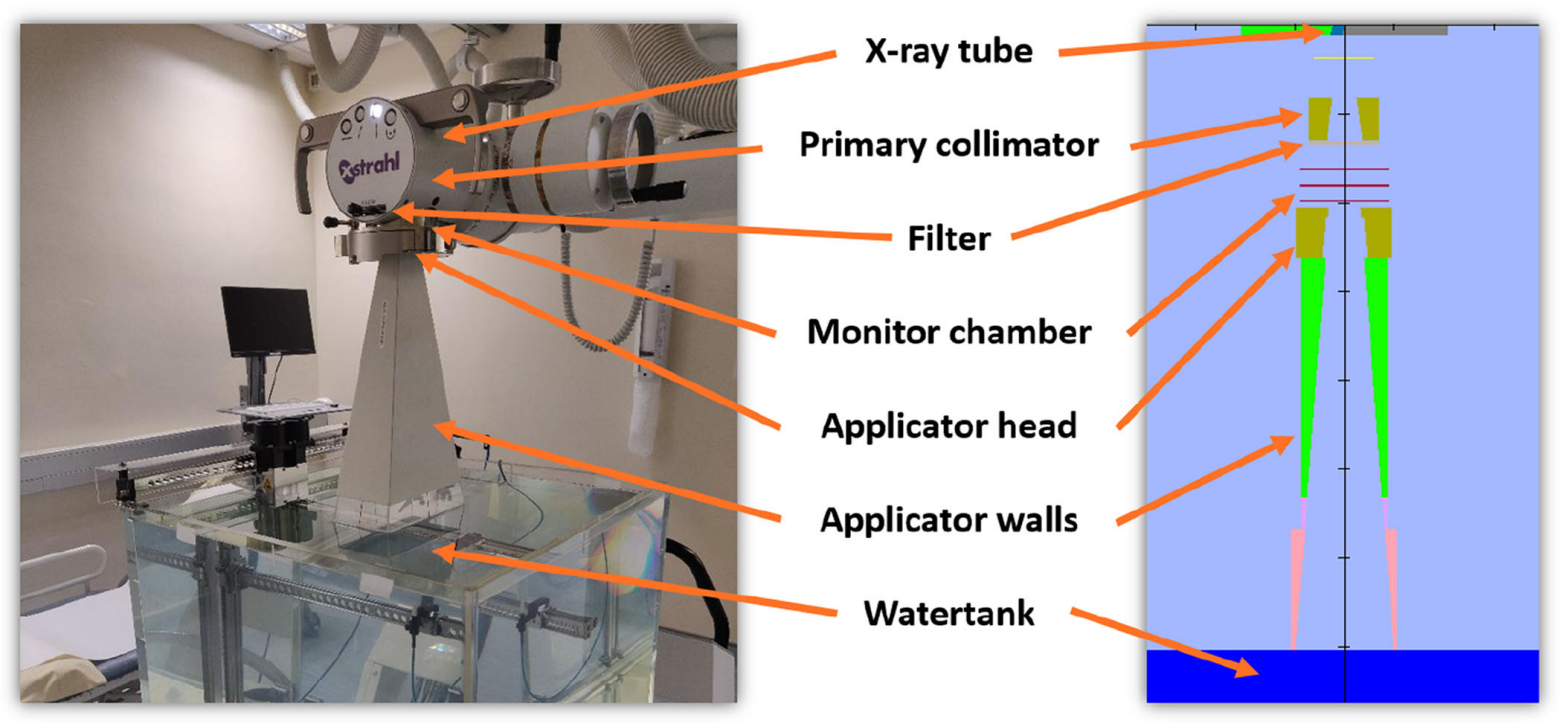
Kilovoltage unit photo and BEAMnrc model of all components

In the second configuration (Figure 1), a water phantom was included at the exit plane of each applicator, where a PHSP was scored to determine the BSF using equation^23^:

(1) $$B_{w}^{\left(w\right)}=\frac{\int_{0}^{\infty }\left[{\left(\frac{d\phi }{dE}\right)}^{\left(0\right)}+{\left(\frac{d\phi }{dE}\right)}^{\left(w\right)}\right]E{\left[\frac{\mu_{\text{en}}}{\rho }\left(E\right)\right]}_{w}dE}{\int_{0}^{\infty }\left[{\left(\frac{d\phi }{dE}\right)}^{\left(0\right)}\right]E{\left[\frac{\mu_{\text{en}}}{\rho }\left(E\right)\right]}_{w}dE},$$

where ${\left[\frac{\mu_{\text{en}}}{\rho }\left(E\right)\right]}_{w}$ is the mass energy absorption coefficient of water, $\frac{d\phi }{dE}$ is the differential fluence and superscripts (0) and (*w*) represent the scoring of the spectrum in air and water, respectively.

The third configuration was used for HVL determination using narrow beam geometry in air. Air kerma K(t) was calculated for an unattenuated beam and HVLs were determined through an iterative process of changing the absorbing material thickness *t* until the following equation^24^ was satisfied:

(2) $$\frac{K(t)}{K(0)}=\frac{\sum_{i=1}^{n}{\left(\frac{\mu_{\text{en}}}{\rho }\right)}_{i}E_{i}\phi_{i}e^{-\left(\mu_{i}^{\text{abs}}-\mu_{i}^{\text{air}}\right)t}\Delta E_{i}}{\sum_{i=1}^{n}{\left(\frac{\mu_{\text{en}}}{\rho }\right)}_{i}E_{i}\phi_{i}\Delta E_{i}}=\frac{1}{2},$$

where $\frac{\mu_{\text{en}}}{\rho }$ is the mass energy absorption coefficient of air, $\mu^{\text{abs}}$ is the linear attenuation coefficient of the absorbing material (Al or Cu), and the product $E_{i}\phi_{i}$ is the energy fluence at a particular spectrum bin.

DOSXYZnrc was used to create a voxellized watertank of size 40×40×30 cm and voxels of size 2×2×2 mm, sufficient to provide full scatter conditions for the largest applicator simulated (20 × 20 cm^2^). PHSP files obtained in the first BEAMnrc configuration were used as inputs for dose to water simulations. The resulting (.3ddose) files were processed by a Python code to extract PDD curves from the surface to a depth of 30 cm and in‐plane and cross‐plane beam profiles at depths of 10, 50, and 100 mm. Simulated output factors were calculated as the ratio of the central axis surface dose to that of a reference applicator for each energy.

The simulated parameters were validated against reference data. A PTW Freiburg GmbH MicroDiamond detector, suitable for measurements in kV energy range,^25^ was used in a PTW BEAMSCAN watertank to obtain PDD curves and beam profiles for all 49 clinical energy–applicator combinations. Reference BSF data were obtained from IPEMB code of practice and its addendum.^12^, ^26^ HVLs and output factors were taken from the commissioning data.

### 2.2 Python applications

**FIGURE 2:**
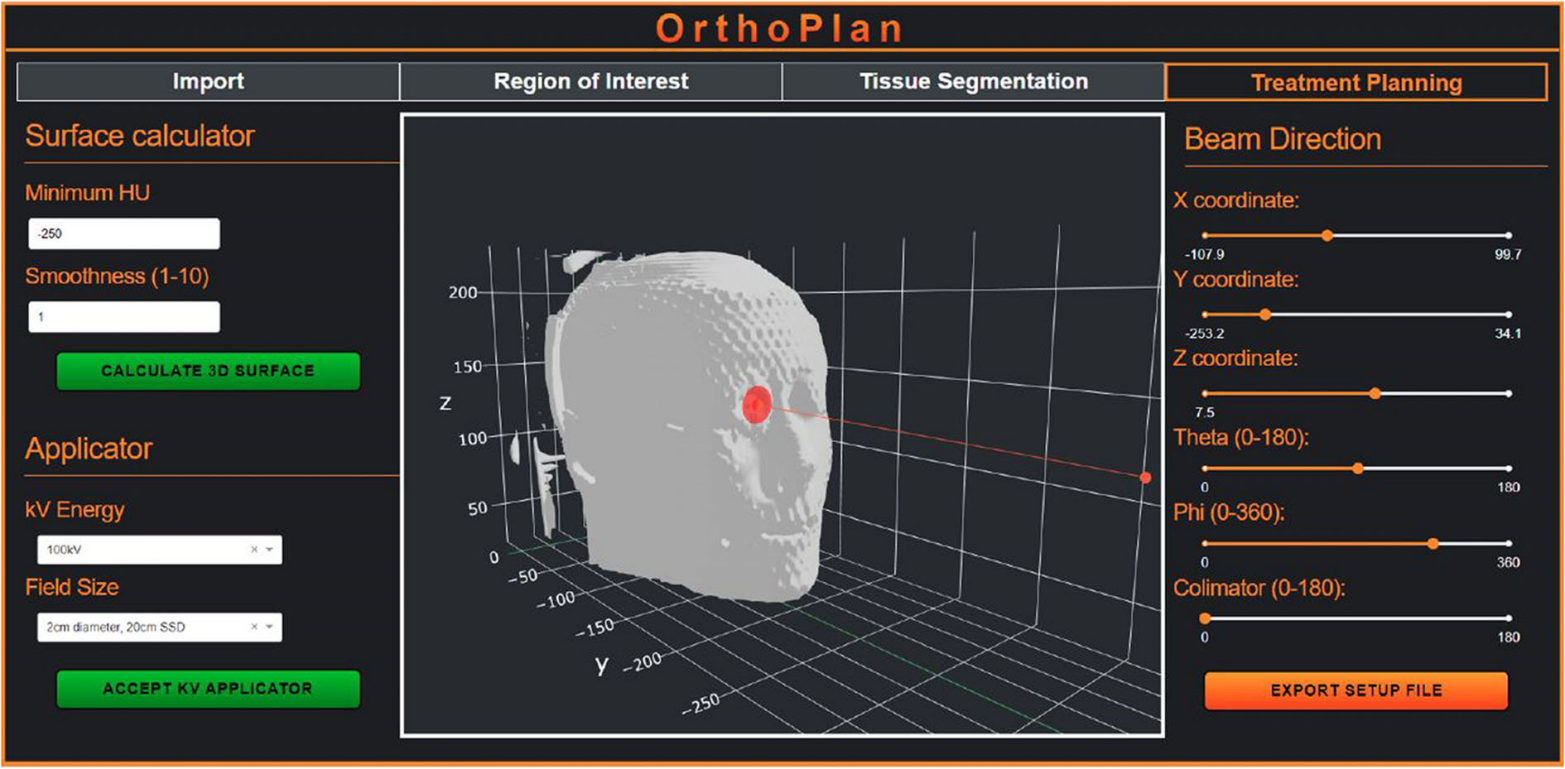
Planning interface of OrthoPlan with a sample patient head scan. The tabs on the top panel reflect the established workflow of this TPI. The left side panel allows the user to triangulate a surface of a given HU value and specified smoothness parameter as well as to select the applicator to be used in the simulation which is then represented by the red plane in the middle 3D interface. The right panel allows the user to modify the position of the applicator exit window plane (red plane) using sliders.

**FIGURE 3:**
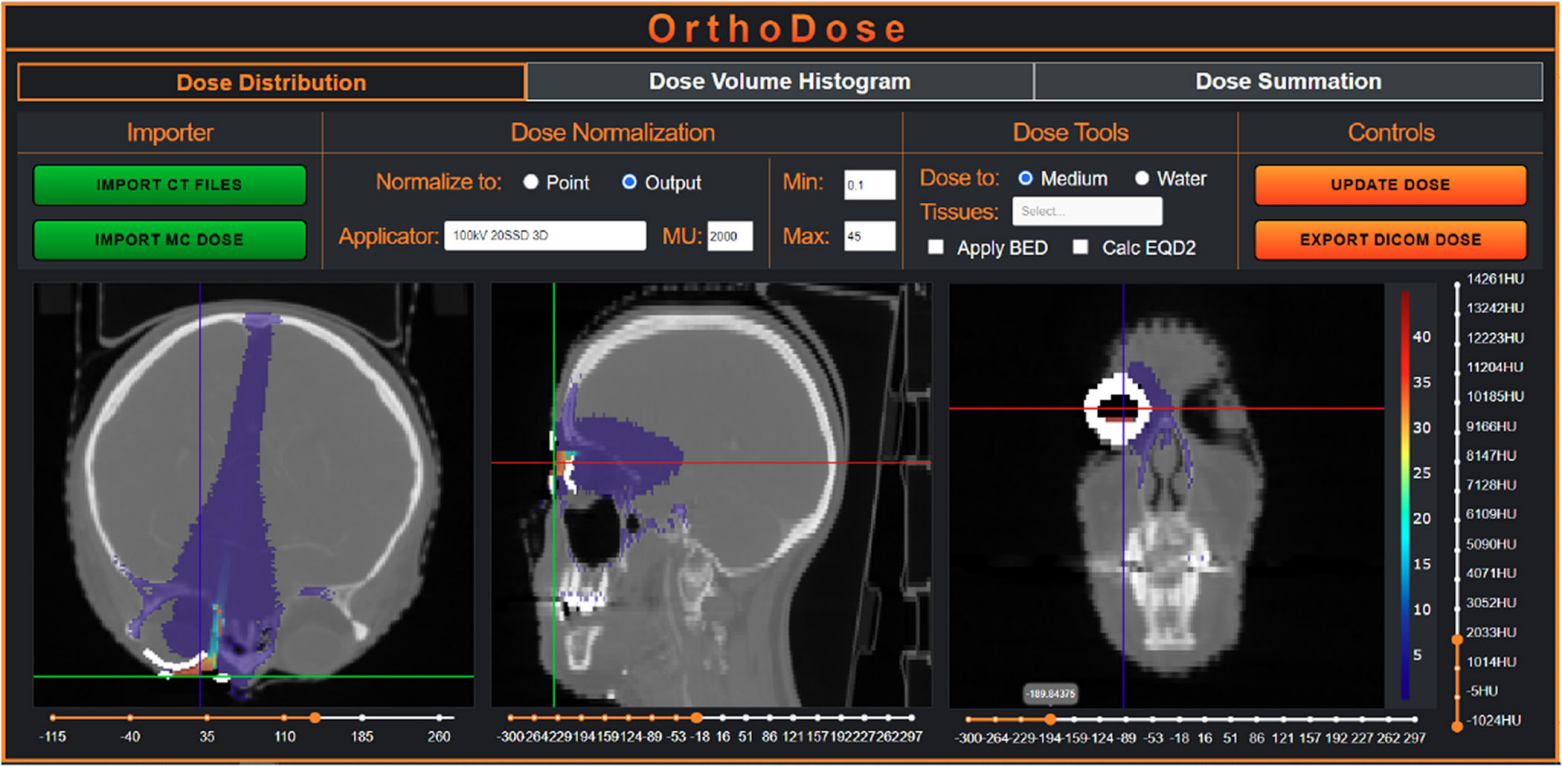
OrthoDose application interface with a sample dose distribution simulated using a lead cutout and an internal gold eyeshield

We developed two Python‐based applications in this work—‘OrthoPlan’ (Figure 2) and ‘OrthoDose’ (Figure 3). OrthoPlan uses ‘pydicom’ module to read DICOM CT dataset and structure files exported from a commercial TPS, such as Eclipse (Varian Medical Systems, Inc.). The HU data are stored in a three‐dimensional array and structures are converted to masked arrays of the same dimensions. With this approach, it is possible to overwrite HU values inside selected structures creating a new DICOM CT set. OrthoPlan allows the user to crop any regions of CT data that are not of interest for the simulations, typically the air surrounding the patient as well as any distant CT slices away from the treatment site. The user is then prompted to select a set of tissues based on the treatment site (head and neck/torso/extremities) and export a DOSXYZnrc phantom file of the appropriate format. The planning interface of OrthoPlan calculates and renders a triangulated phantom surface using ‘skimage.measure.marching_cubes’ and ‘plotly.graph_objs.Mesh3d’ functions, respectively. A 3D interactive Plotly graph is then used to set up the selected beam in spherical polar coordinates consistent with the DOSXYZnrc input file which is then automatically generated. The exported phantom and input files are then used in DOSXYZnrc MC simulation producing a dose file.

OrthoDose reads the patient DICOM CT data as well as the dose file. The dose distribution is then normalized to a point or the output of the kV unit using:

(3) $$D_{\text{absolute}}=\frac{D_{\text{SimPhantom}}}{D_{\text{SimWater}}}\times O\times \text{MU},$$

where $D_{\text{SimPhantom}}$ is the dose from the current phantom simulation (Gy/particle), $D_{\text{SimWater}}$ is the dose at the central surface voxel using the same PHSP in a watertank simulation (Gy/particle), *O* is the kV unit output for the current applicator (Gy/MU), and MU is the prescribed monitor units. Due to the nature of MC, the reported dose is, by default, dose to medium. The absolute dose distribution can then be examined in OrthoDose, where a dose volume histogram can also be calculated for a structure set. The dose distribution can be exported as a DICOM dose file to a clinical TPS, such as Eclipse, where it can be reviewed using all available tools. This allows it to be compared and/or summed with other dose distributions aiding in clinical decision making.

### 2.3 Tissue segmentation

**FIGURE 4:**
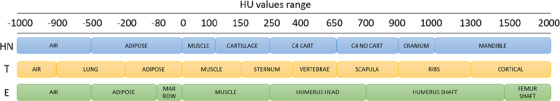
Tissue segmentation ranges for head and neck (HN), torso (T), and Extremity tissue groups (E)

PEGS4 (preprocessor for EGS) code was used to generate material data for the DOSXYZnrc CT phantom simulations. Elemental composition of a range of tissues was taken from ICRU report 44 as well as other literature.^10^, ^27^ Density correction files were generated using the National Institute of Standards and Technology (NIST) tool ESTAR.^28^ EGSgui user code was then used to compile a PEGS4 materials file. Tissues were grouped in OrthoPlan based on anatomical locations in order to span the HU values with appropriate tissue types. Tissue segmentation ranges are given in Figure 4. Air was assigned to all HU values lower than the minimum HU of first tissue in each group and heavy metals such as lead and gold were assigned to the high end of the HU value range in each group. Air, lead, and gold composition and density were taken directly from the default EGS PEGS4 database. The tissue groups can be edited and further optimized over time if there will be a clinical need for other tissue types. HU to mass density curve (derived using a CIRS electron density phantom) was used to interpolate the physical density of each voxel using its HU value.

### 2.4 End‐to‐end testing

**FIGURE 5:**
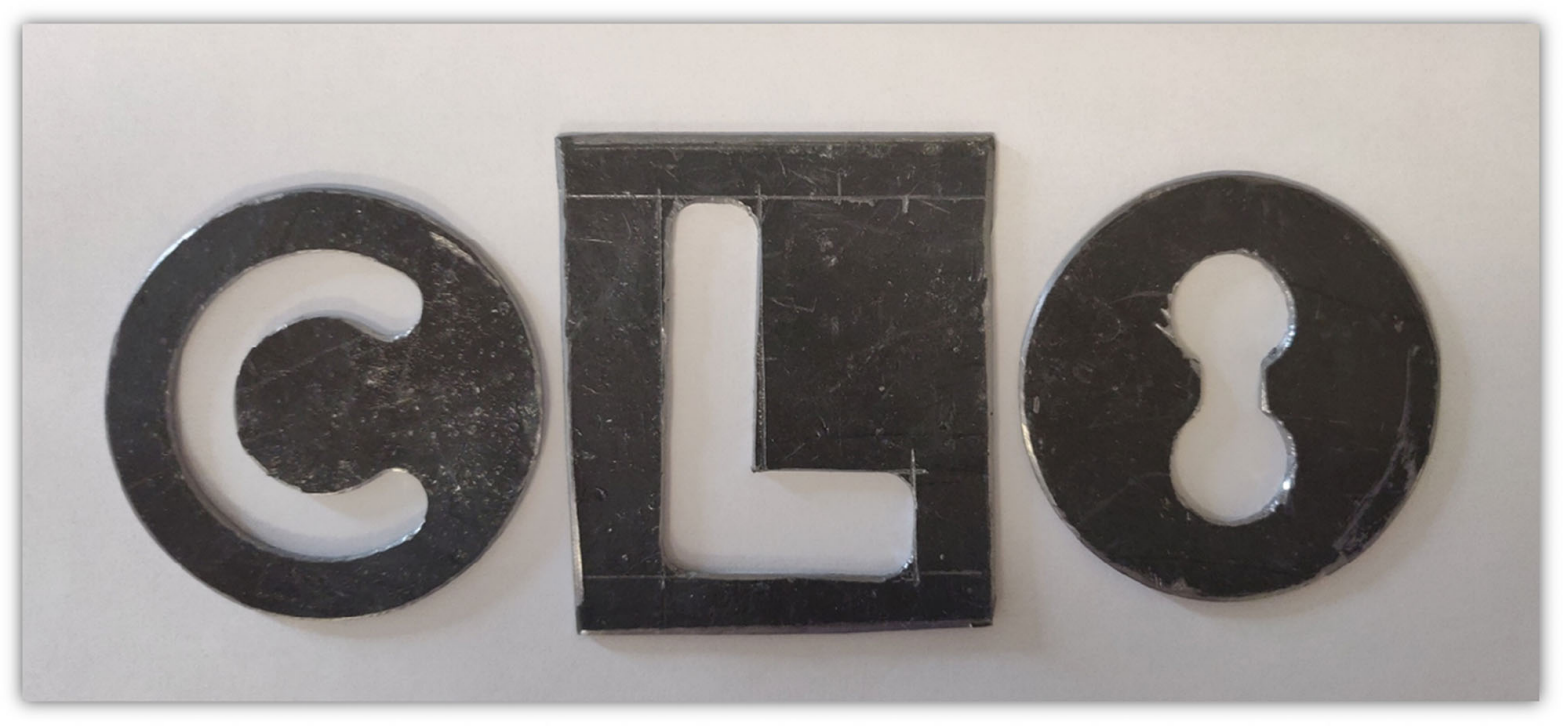
Schematic images of lead cutouts used in end‐to‐end testing

**FIGURE 6:**
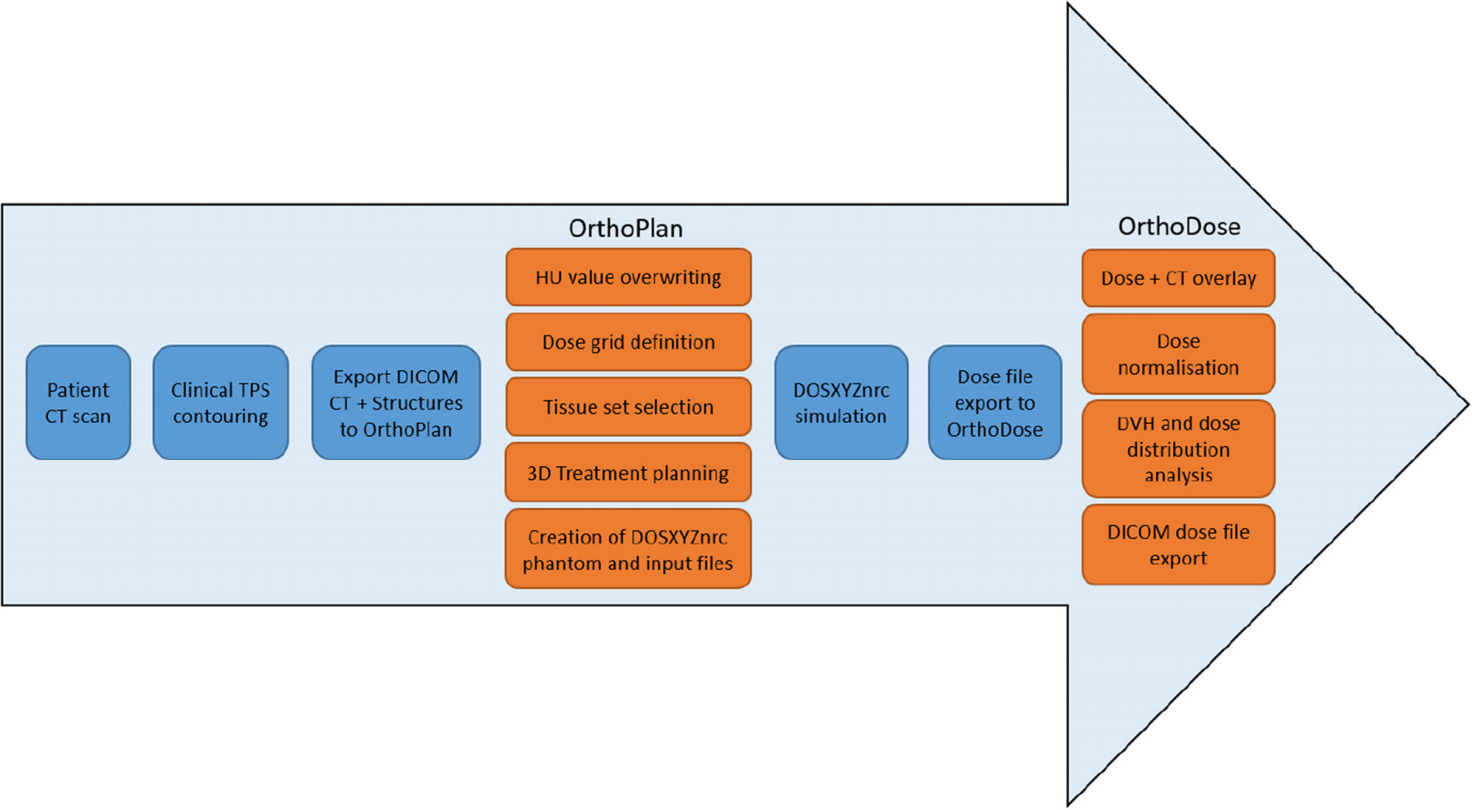
Proposed clinical workflow for kilovoltage treatment dose calculation

Lead cutouts shown in Figure 5, representing challenging geometries, were used in end‐to‐end testing of cutout simulations. EBT3 GafChromic film was calibrated using a standard procedure in the dose range 0–3 Gy. Test film was irradiated at 1 cm depth in CIRS plastic water phantom with beams shaped using the aforementioned cutouts. This setup was replicated in a simulation using the proposed clinical workflow (Figure 6) and the absolute dose distribution was calculated using the delivered monitor units. FilmQAPro software (Ashland, Bridgewater NJ, USA) was used to compare the measured and simulated absolute dose distribution using gamma analysis with criteria of 3%/2 mm with a 20% and a 50% thresholds.

A water phantom with three compartments was created to further verify dose in heterogeneous media through comparison of measured and calculated data. Compartments were filled with water and material analogs for adipose (vegetable oil) and muscle (mixture of flour and water) tissues. PTW 0.3cc Semiflex detector was used to measure ionization in the center of each compartment for 100 monitor units using 100 kV beam with a 4 cm diameter applicator. Dose to water calibration coefficient was derived using

(4) $$N_{\text{dw}}=\frac{D_{w}^{\text{MC}}}{M_{w}\times {\mu_{\text{en}}}_{\text{air}}^{\text{water}}}$$

where $D_{w}^{\text{MC}}$ is the MC calculated absolute dose in the center of the water compartment, $M_{w}$ is the charge reading in the center of the water compartment, and ${\mu_{\text{en}}}_{\text{air}}^{\text{water}}$ is the mass energy absorption coefficient ratio of water to air weighted over the beam spectrum. $N_{\text{dw}}$ was then used to calculate dose to water in adipose and muscle compartments using the charge readings. Mass energy absorption coefficients of tissue analogs were estimated using NIST XCOM database and were used to convert from dose‐to‐water to dose‐to‐medium.

## 3 RESULTS

### 3.1 Model validation

**FIGURE 7:**
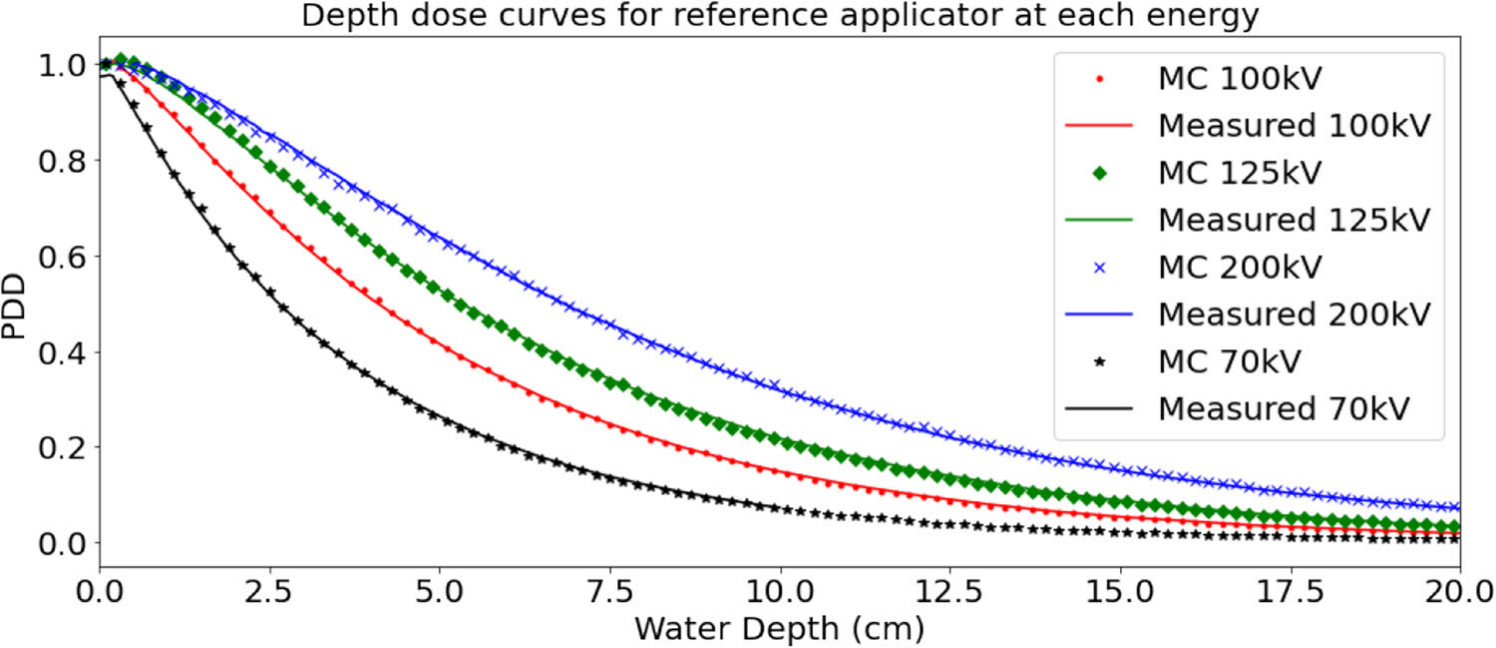
Sample percentage depth dose (PDD) curve comparison for reference applicator at each beam energy. Reference applicators are 10 cm diameter circle at 30 cm source‐to‐surface distance (SSD) for 70, 100, and 125 kV beams, and 10 × 10 cm^2^ at 50 cm SSD for 200 kV beam

Measured and simulated PDD curves were normalized to the surface dose. Point‐by‐point percentage difference for all PDDs agreed to within 2%. Sample measured and simulated PDD curves for reference applicators can be seen in Figure 7.

**FIGURE 8:**
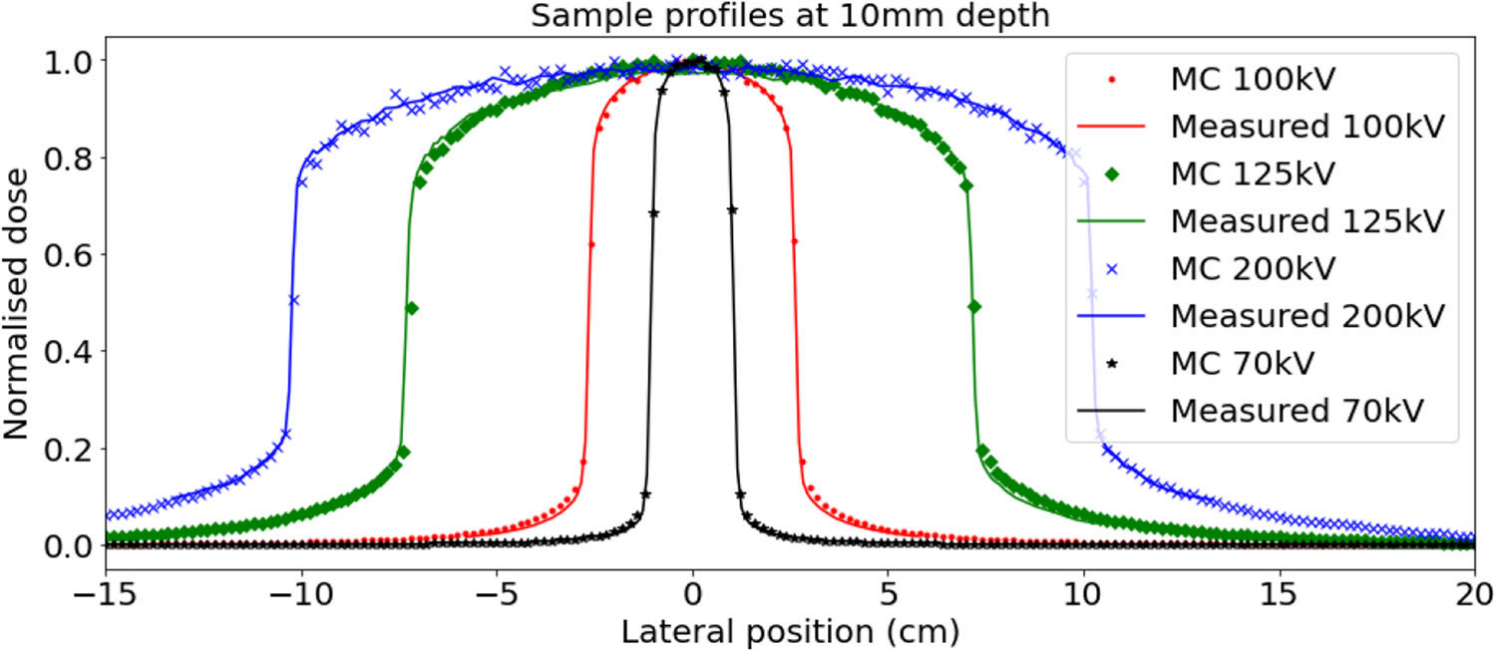
Sample beam profile comparison for a set of field sizes with different energies

Beam profiles were normalized to the area under the curve within the 80% profile width, which is the main clinical region of interest. Sample profiles can be seen in Figure 8. For each profile, point‐by‐point percentage differences between the measured and simulated values were calculated and summarized into mean, max, and standard deviation values. The mean percentage differences were of the order of 0.01% demonstrating good distribution of simulated points about the measured profile. The standard deviation values represent the level of agreement between simulated and measured data and were of the order of 2% for the majority of the profiles. The maximum values represent a single simulated point in the profile with the largest deviation from the measured point. For all profiles, the maximum point percentage differences were within 4%.

The HVL comparison can be seen in Table 2. Good agreement was found between simulated and measured HVL values, with a maximum absolute difference of 0.445 mm of aluminum for the 125 kV beam which is within the acceptable clinical tolerance of 10%.

**TABLE 2:** Measured and MC calculated HVL values for each of the clinical energy beams

**kVp**	**HVL MC**	**HVL measured**	**% Difference**
70	1.99 mm Al	2.15 mm Al	7.4%
100	4.15 mm Al	4.25 mm Al	2.4%
125	7.68 mm Al	8.125 mm Al	5.5%
200	1.83 mm Cu	1.85 mm Cu	1.1%

Abbreviations: HVL, half‐value layers; MC, Monte Carlo.

**FIGURE 9:**
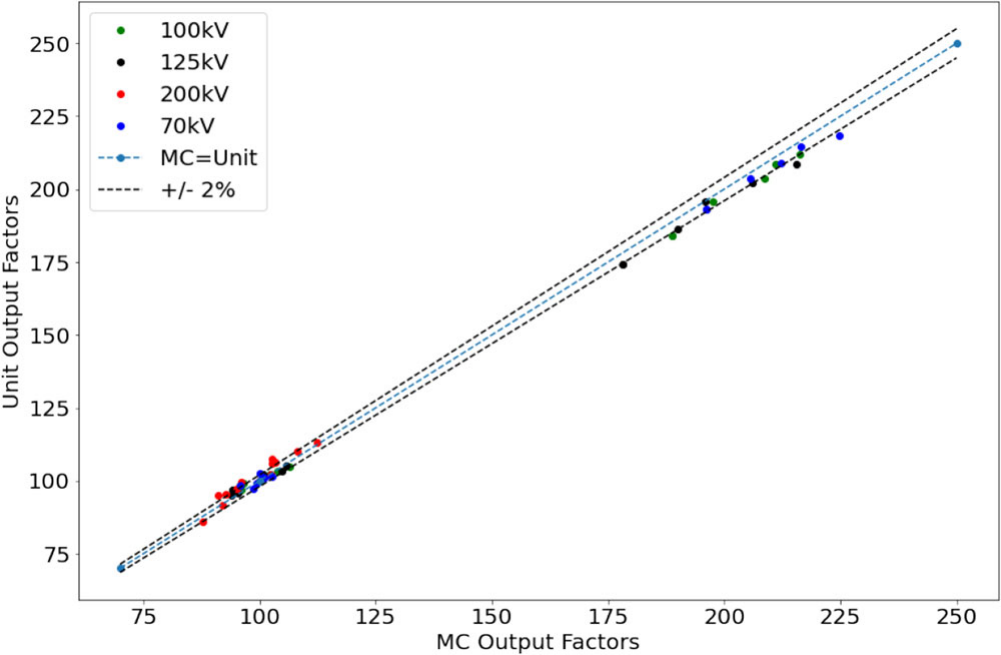
Measured vs. Monte Carlo (MC) simulated output factors

Simulated BSF in Tables 3 and 4 were in good agreement with IPEMB data with the majority agreeing to within 1.5%, and a maximum difference of 2.9% for the 200 W setup (200 kV, 15 × 15 cm, 50 cm SSD). As seen in Figure 9, the simulated output factors were on average −0.1% ± 2% (*k*=1) different from the measured output factors showing a good relationship across all applicators modeled.

**TABLE 3:** MC calculated and IPEMB BSF for 70 and 100 kV applicators

App.	MC	IPEMB	% Diff
70A	1.124 ± 4.7%	1.112	1.05
70B	1.155 ± 4.7%	1.143	1.05
70C	1.173 ± 2.1%	1.165	0.7
70D	1.192 ± 2.1%	1.188	0.3
70E	1.215 ± 3.2%	1.202	1.11
70F	1.23 ± 3.2%	1.228	0.16
70G	1.239 ± 3.2%	1.248	−0.73
70H	1.268 ± 3.2%	1.269	−0.1
70I	1.226 ± 3.2%	1.212	1.19
70J	1.239 ± 3.2%	1.224	1.19
70K	1.27 ± 3.2%	1.254	1.26
70L	1.266 ± 3.2%	1.264	0.2
100A	1.12 ± 3.3%	1.121	−0.11
100B	1.165 ± 3.3%	1.165	−0.01
100C	1.206 ± 1.5%	1.202	0.35
100D	1.233 ± 1.5%	1.231	0.12
100E	1.26 ± 1.5%	1.256	0.34
100F	1.302 ± 2.2%	1.303	−0.09
100G	1.328 ± 2.2%	1.333	−0.34
100H	1.37 ± 2.2%	1.375	−0.38
100I	1.284 ± 2.3%	1.275	0.71
100J	1.297 ± 2.3%	1.296	0.08
100K	1.351 ± 2.3%	1.345	0.47
100L	1.372 ± 2.3%	1.364	0.58

Abbreviations: BSF, backscatter factors; MC, Monte Carlo.

**TABLE 4:** MC calculated and IPEMB BSF for 125 and 200 kV applicators

**App**.	**MC**	**IPEMB**	**% Diff**
125A	1.124 ± 2.7%	1.109	1.36
125B	1.167 ± 2.7%	1.154	1.12
125C	1.212 ± 1.2%	1.198	1.15
125D	1.239 ± 1.2%	1.236	0.27
125E	1.272 ± 1.2%	1.266	0.5
125F	1.32 ± 1.8%	1.322	−0.18
125G	1.364 ± 1.8%	1.364	−0.02
125H	1.415 ± 1.8%	1.417	−0.14
125I	1.3 ± 1.8%	1.288	0.92
125J	1.325 ± 1.8%	1.314	0.83
125K	1.394 ± 1.8%	1.380	1.02
125L	1.419 ± 1.8%	1.404	1.08
200M	1.171 ± 4.1%	1.171	0.04
200N	1.217 ± 4.2%	1.21	0.56
200O	1.247 ± 4.2%	1.233	1.12
200P	1.281 ± 4.2%	1.26	1.7
200Q	1.268 ± 4.2%	1.249	1.5
200R	1.288 ± 4.2%	1.283	0.43
200S	1.318 ± 4.1%	1.303	1.19
200T	1.35 ± 4.1%	1.335	1.12
200U	1.328 ± 4.1%	1.307	1.57
200V	1.354 ± 4.1%	1.334	1.54
200W	1.414 ± 4.1%	1.374	2.9
200X	1.372 ± 4.1%	1.351	1.57
200Y	1.448 ± 4.1%	1.423	1.73

Abbreviations: BSF, backscatter factors; MC, Monte Carlo.

### 3.2 End‐to‐end testing

**FIGURE 10:**
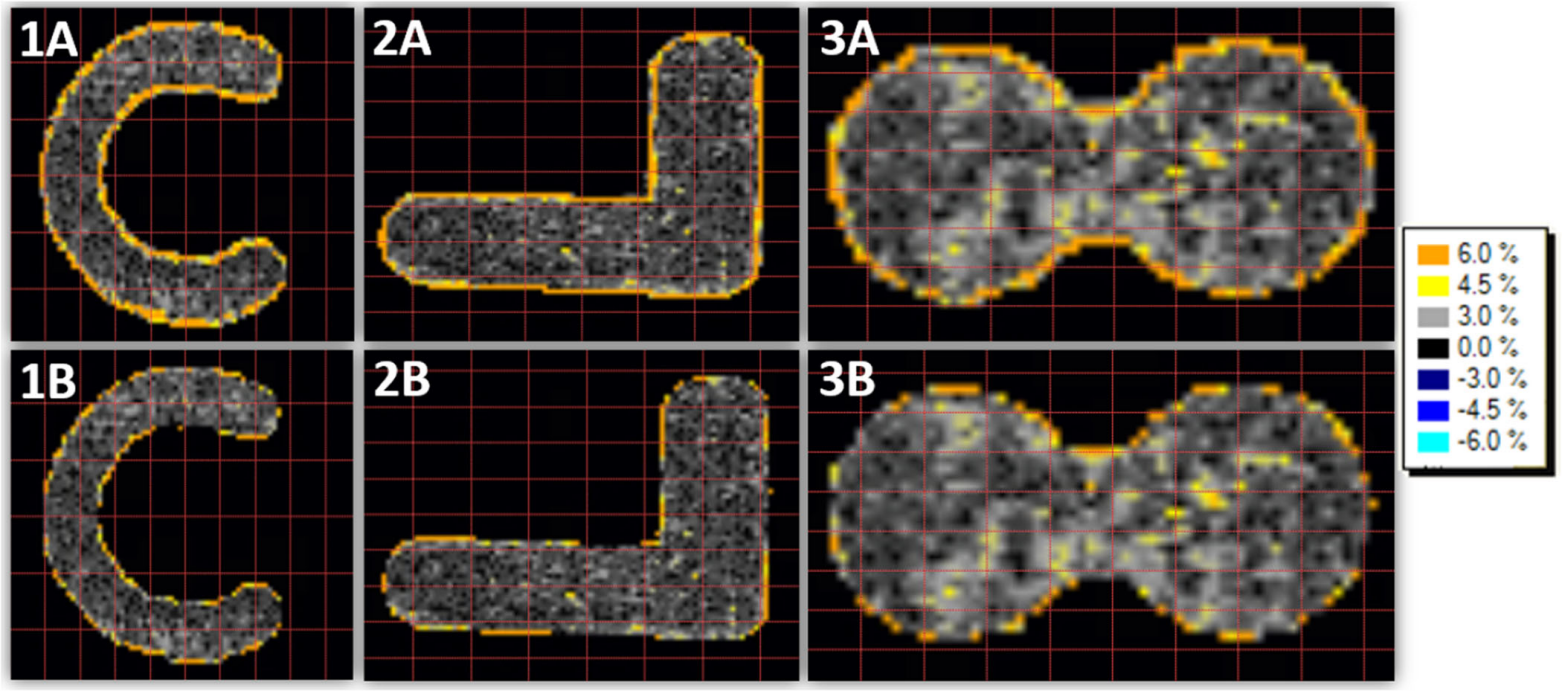
Gamma map for (a) 20% threshold and (b) 50% threshold

**FIGURE 11:**
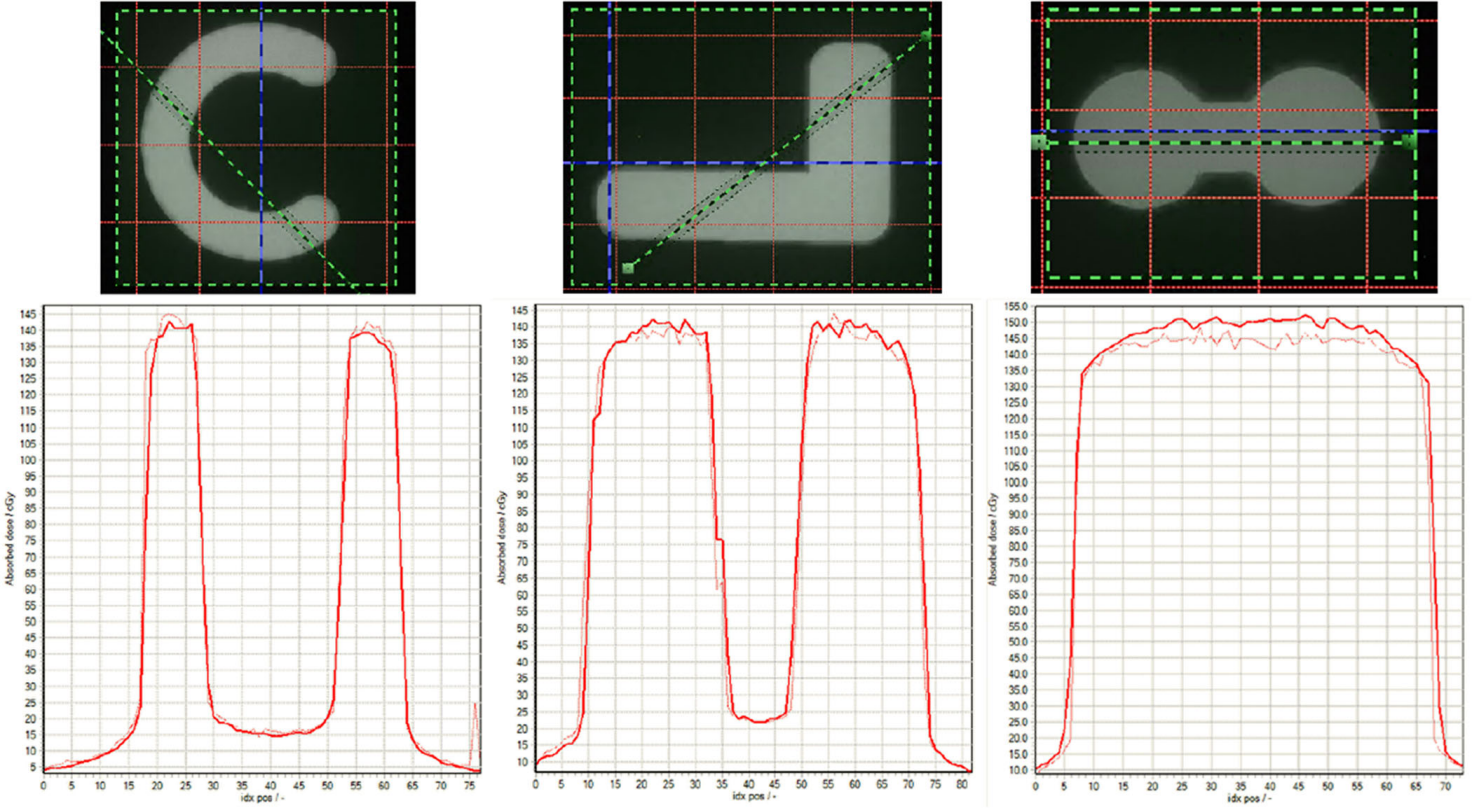
Solid line (simulated) and dotted line (measured) sample profiles from measured and simulated absolute dose distributions

The red color channel was used for the analysis of irradiated test film as it had the largest dynamic range. Absolute dose gamma analysis results can be seen in Table 5. Visual inspection of the gamma distribution (Figure 10) revealed majority of in‐field points agreeing to within 3% with larger differences on the edges of the fields. Sample dose profiles (Figure 11) showed good qualitative agreement within 5 cGy (3%) despite the noise present in the profiles.

**TABLE 5:** Gamma analysis with different criteria for the end to end testing of absolute dose distributions generated using custom lead cutouts

**Gamma criteria**	**‘C’ passing**	**‘L’ passing**	**‘8’ passing**
3%/2 mm 20% threshold	80.1%	86.4%	82.8%
3%/2 mm 50% threshold	89.8%	92.9%	87.8%

MC doses in heterogeneous phantom were calculated to 1% uncertainty. Dose‐to‐water was taken as a reference and used to calculate the calibration coefficient $N_{\text{dw}}=0.542$. Calculated and measured dose differences in Table 6 were within 4% for adipose and 7% for muscle tissues.

**TABLE 6:** Measured and calculated dose to heterogeneous phantom tissues

Medium	MC $D_{m}$ (Gy)	$M_{\text{avg}}$ (nC)	${\mu_{\text{en}}}_{\text{air}}^{\text{med}}$	Meas. $D_{m}$ (Gy)	Diff
Water	0.63	1.135	1.0239	0.63	0%
Adipose	0.44	1.33	0.6346	0.457	4%
Muscle	0.54	1.072	0.9962	0.579	7%

Abbreviation: MC, Monte Carlo.

## 4 DISCUSSION

### 4.1 Model validation

Forty‐nine clinical setups were modeled and validated using beam profiles, depth dose curves, HVL, BSF, and output factors. The initial model parameters resulted in a good level of agreement which was comparable to that reported in other studies and was within the tolerances that would normally be accepted in a clinical TPS.^29^ Therefore, no modifications were made in an attempt to further improve the model agreement.

The incident electron beams were modeled as monoenergetic with no Gaussian distribution of energies. Although in reality the accelerating voltage will have small fluctuations, the exact values are not known and hence cannot be used to accurately model the Gaussian spread. Also, once the monoenergetic electron beam hits the tungsten target, inelastic scattering interactions dominate resulting in an immediate broadening of the single monoenergetic peak to a Gaussian spread of energies.

Some previous studies showed HVL matches within 2.3%–5.0%, while others reported a mismatch of up to 5.2 mm Al.^14^, ^24^, ^30^, ^31^, ^32^ In this work, the HVLs matched to within 0.5 mm for all simulated beams with the small differences attributed to the impurities present in the aluminum sheets. Improved HVL matching was likely due to an accurate match of the beam filtration which dictates the beam spectrum which was used to calculate the HVLs.

Several groups have validated their models using PDDs and beam profiles with reported agreement within 6% for PDDs and 10% for beam profiles at depth.^14^, ^15^, ^16^, ^31^, ^32^, ^33^ In this work, the PDDs agreed to within 2% for all points across all simulated beams demonstrating a good match of beam quality. This agreement could be attributed to a good match of the source electron beam and photon beam filtration. All beam profiles in this work were examined in the 80% field width showing a good match to within 4% at all points. Out of field points were not quantitatively compared but nonetheless demonstrated a good agreement with reference data which can be attributed to the good match of the modeled applicator wall geometry.

This work extended the model validation to include BSF and output factors which was not done previously. The simulated BSFs were in good agreement with published IPEM data to within 2.9% across all simulated applicators. These results also serve as an independent check of IPEM data which are used by other centers. Simulated output factors matched to within 3% with the reference data for the 49 energy–applicator combinations studied.

### 4.2 Tissue segmentation

Previous work showed that fine tissue segmentation is of high importance for accurate kV radiotherapy dosimetry.^9^, ^10^ In this work, tissue compositions were taken directly from the literature^10^, ^27^ rather than generated via interpolation of published data.^10^ Tissues were grouped based on their anatomical location resulting in each HU value range being assigned a single unambiguous tissue type. Using this approach, the relative dose deposited in different tissues was in agreement with literature,^9^ providing confidence in simulations which cannot be compared to explicit measurements of dose.

### 4.3 Python TPI

With no commercial TPS available for kV radiotherapy, in‐house solutions have to be developed. In contrast to previous publications, using C++ and MATLAB codes, Python was used to develop the TPI in this work extending the functionality of previous publications^16^, ^19^ and opening up the possibility of sharing the code with other users. The ability to overwrite structure HU values can be used to introduce gold and lead internal shields as well as lead cutouts which are commonly used clinically to conform the beam to the exact tumor shape and spare organs at risk. This functionality can also be used to account for CT image artifacts.

Commercial TPSs define LINAC beam geometry using couch, gantry, and collimator angles which are then used for treatment setup. In contrast, kV units are manipulated manually to set up non‐co‐planar beams. TPI developed in this work incorporates a 3D interactive planning interface that allows visualization of the non‐co‐planar beam setup, simplifying the treatment planning process.

Another feature of the Python framework developed in this study is the absolute dose scaling by normalizing the dose based on the output of the kV unit in reference water geometry which is the approach implemented in commercial TPSs.

Although the resultant dose distribution and dose–volume histograms can be analyzed in OrthoDose, a DICOM dose file can also be exported to a commercial TPS, where it can be compared to other dose distributions using an extensive set of tools. It can also be stored in patient records for future reference.

### 4.4 End‐to‐end testing

Despite the noise present in measured and simulated data using lead cutouts, the absolute dose agreement was satisfactory within 3% at most points in and out of field. This agreement validates the absolute dose normalization approach. Sample profile agreement also demonstrates accurate dosimetry out of field (behind the lead cutout). Larger dose differences were observed on the edges of the cutout‐shaped fields which can be attributed to the cutout contouring inaccuracy and voxelization of the smooth physical cutout geometry for the simulation. These differences were only observed within 1 mm of the cutout edges which is representative of the tolerance typically used for LINAC multi‐leaf collimator quality assurance and hence acceptable as uncertainty in this work. The in‐field gamma dose differences can be attributed to the slight difference in simulated field size (due to the above limitations) and hence more phantom scatter contributing to the dose in the center of the field. To the best of our knowledge, custom‐shaped kV dose distribution gamma analysis was not previously published and hence no comparison can be made to other work. Sample patient simulations took approximately 10 h to achieve an uncertainty below 1.5% in the region of interest. Although the simulation takes significantly longer than a typical commercial TPS dose calculation we note that a full MC calculation is performed and the ‘overnight’ time frame is acceptable in our facility.

Although MC calculations show differential dose absorption in different tissues, dose‐to‐medium cannot be measured, and hence validated, directly. In this work, we have measured air ionization in adipose and muscle tissue surrogates and converted charge readings to dose‐to‐medium using the ratio of mass energy absorption coefficients obtained from NIST XCOM database. The dose reduction seen in the MC simulations of the heterogeneous volumes showed a similar trend to measured dose estimates and agreed to within 4% for adipose and 7% for muscle, despite several approximations being made in the process. Further investigations into measuring dose‐to‐medium were outside the scope of this work but do require attention. In current clinical practice, dose to all tissues is assumed to be that of water. This work, among others,^9^, ^10^ highlights the importance of including tissue segmentation in kV dose determination.

The proposed treatment planning workflow in this work will not be applied to all standard treatment cases as simulations require CT images which are not typically acquired for kV treatments. Our framework will be most useful for patients undergoing another course of radiotherapy requiring a CT scan. It can also be used as a research tool to simulate complex treatment setups for educational purposes on sample CT images.

## 5 CONCLUSION

EGSnrc applications: BEAMnrc, DOSXYZnrc, and BEAMDP were used to create models of all clinically used kV unit beams in our facility and to determine a full set of parameters used in model validation—PDD, profiles, HVL, BSF, and output factors. Xstrahl 200 unit model was created and extensively validated in this work, extending the range of energies, applicators, and validation parameters previously published. For CT phantom simulations using DOSXYZnrc code, tissue segmentation was performed based on published elemental compositions of common tissue types as well as anatomical grouping to segregate tissues of similar density but different composition.

The TPI developed in this work extends the functionality of previously developed frameworks allowing beam modification using lead cutouts and shields, commonly present in kV treatments. The absolute dose calculation functionality was validated using gamma analysis in an end‐to‐end testing of custom‐shaped fields as well as ion chamber measurements in a heterogeneous phantom, which was not previously published. This functionality allows clinical examination of dose distributions around organs at risk which was not possible previously using manual point dose calculations. The 3D planning interface of the TPI developed in this work provides a user‐friendly environment to set up non‐co‐planar beams used in kV treatments. Python, being the language used to develop the TPI, opens the possibility to share the code with other users due to its growing use in the medical physics community.

Future work will involve implementation of a radiobiological effectiveness (RBE) factor for kV energy beams as well as calculating biologically effective dose (BED) distributions in our TPI. A library of sample patient dose distributions will be accumulated over time and will serve as a useful tool for educational purposes as well as a reference for similar treatments in the future.

## AUTHORs' CONTRIBUTION

Mihails Nikandrovs: conceptualization, methodology, software, validation, formal analysis, visualization; writing—original draft; Brendan McClean: writing—review and editing, conceptualization, supervision; Laura Shields: conceptualization, methodology; Patrick McCavana: conceptualization, software, formal analysis; Luis León Vintró: writing—review and editing.

## CONFLICT OF INTEREST

The authors declare that there is no conflict of interest that could be perceived as prejudicing the impartiality of the research reported.
